# How to Promote Ethnic Village Residents’ Behavior Participating in Tourism Poverty Alleviation: A Tourism Empowerment Perspective

**DOI:** 10.3389/fpsyg.2020.02064

**Published:** 2020-08-18

**Authors:** Jianchun Yang, Jialian Wang, Lei Zhang, Xiaohong Xiao

**Affiliations:** School of Business Administration, Guizhou University of Finance and Economics, Guiyang, China

**Keywords:** tourism empowerment, participation behavior, participation willingness, participation ability, tourism poverty alleviation

## Abstract

Local villagers are regarded as the main part of tourism development in the ethnic village, their participation in tourism poverty alleviation has a vital impact on sustainable tourism development and poverty reduction. Based on the planned behavior theory and capacity approach theory, we investigated the influence of tourism empowerment on ethnic village residents’ behavior participating in tourism poverty alleviation, especially focusing on the mediating effect of participation willingness and the moderating effect of participation ability. We took Zenlei Village in Sandu County of Guizhou Province in China as research subjects and analyzed 239 valid samples through the structural equation model and hierarchical regression. The results indicate that: (a) Tourism empowerment has a remarkable positive effect on participation behavior. (b) Participation willingness plays a partial mediating role between tourism empowerment and participation behavior. (c) Participation ability positively moderates the positive relationships between tourism economic empowerment, tourism psychological empowerment, and participation willingness. That is, the positive relationships between tourism economic empowerment, tourism psychological empowerment, and participation willingness would be stronger when villagers have higher participation ability. Whereas, participation ability has not yet played a moderating role between tourism social empowerment, tourism political empowerment, and participation willingness.

## Introduction

Tourism is characterized by high degree of industrial correlation, low threshold of employment, strong comprehensive driving force and great radiation traction, these traits have increasingly highlighted tourism’s role in promoting regional economic development and helping the poor out of poverty, thus, it has become one of the important forms of anti-poverty. Tourism poverty alleviation is based on the principle of “poverty alleviation,” the development of tourism as the strategies and approaches, the anti-poverty and elimination of poverty of vulnerable groups as the core goal, the premise of economic benefits, the comprehensive development of poor communities as the content, the development of poor population as the core, the positive social change as its ultimate goal (Zhou, 2002). Tourism poverty alleviation is not exactly about expanding the size of the industry as it is about offering opportunities to the poor to gain economic and other livelihood benefits, or to participate in decision-making (Ashley et al., 2001). Due to its remote location, inconvenient transportation, low level of social development, less external interference and influence on natural landscape and cultural customs, ethnic villages, poor areas, and areas rich in tourism resources have a high degree of overlap.

In the context of targeted poverty alleviation, the characteristics of “blood transfusion” and “blood production” of tourism poverty alleviation make itself play an increasingly prominent role in driving the development of ethnic villages and poverty reduction of poor villagers. Residents of ethnic villages, as the resource provider, industry operator, and activity beneficiary of tourism poverty alleviation, also provide an indispensable endogenous force for tourism poverty alleviation (Fotiadis et al., 2016). Ethnic village tourism poverty alleviation cannot be separated from the participation of residents, which is not only effectively safeguard residents’ rights and interests (Hwang et al., 2012), but also help protect the resources and environment (Sebele, 2010) and implement relevant policies on tourism development (Lamberti et al., 2011).

However, due to the imbalance of social development in ethnic villages, the villagers seem to be in a disadvantaged position (Li et al., 2016). A series of problems such as low participation enthusiasm and frequency of residents occurs in the process of tourism poverty alleviation in ethnic villages, leading to a strange circle of “more help, more poverty.” The participation of residents is related to the sustainable development of tourism (Murphy, 1988; Sharpley, 2014; Khalid et al., 2019; Shen et al., 2019) and the vital interests of residents (Hwang et al., 2012). During the critical period of China to eradicate absolute poverty and secure a decisive victory in building a moderately prosperous society in all respects, how to inspire ethnic village residents to participate in tourism poverty alleviation and improve the quality and effectiveness of tourism poverty alleviation has become an urgent issue for industry and academia (Yang, 2017).

Based on the systematic review of both of domestic and abroad researches, we find that fewer scholars explored the affecting factors of residents’ participatory tourism behavior through empirical researches. In a few relevant articles, the researchers mainly proved that ecotourism cognition (Huang et al., 2014), perceived justice (Xu et al., 2015), participation opportunities (Zhang, 2015), individual characteristics (Simon, 2016), and attitudes to tourism impacts (Ribeiro et al., 2017) have significant effects on residents’ participatory tourism behavior, but few scholars explored the influence mechanism of residents’ participatory tourism behavior from the perspective of empowerment. Community participation faces various challenges and obstacles (Stone and Stone, 2011), such as operational, structural, and cultural barriers (Tosun, 2000; Dogra and Gupta, 2012). The structural barriers generating by power relations are regarded as the main obstacles for the community to participate in tourism (Weng and Peng, 2010). If the rights of community residents are not guaranteed or even deprived in tourism development, other obstacles for poor residents to participate in tourism cannot be effectively solved (He and Li, 2019). Tourism empowerment emphasizes giving residents personal rights, realizing the balance of power relations, improving the enthusiasm of community residents to participate in tourism development, and paying attention to the empowerment of community residents. The essence of tourism empowerment is to enhance residents’ rights in tourism development, break the unbalanced power relationships, and stimulate better development of communities and tourism (Zuo and Bao, 2008). Thus, one aim of this study is to test the influence of tourism empowerment on ethic village residents’ participation behavior in tourism poverty alleviation.

In the past, most of the researches on the function mechanism of tourism empowerment were theoretical and static studies, lacking of corresponding quantitative evaluation studies, and the empirical studies involving mediating variables were relatively few. During the process of tourism development in ethnic communities, community empowerment is an effective way to promote the willingness of residents to participate in tourism exchange (Liu and Li, 2016). Meanwhile, the theory of planned behavior holds that individuals’ behavior is directly caused by their behavioral intention which is influenced by individual behavioral attitude, subjective norms, and perceived behavioral control (Ajzen, 1991). In another word, residents’ participation willingness directly affects their participation behavior. Although some literatures have revealed the influence of tourism empowerment on residents’ participation willingness or residents’ participation willingness on their participation behaviors (Stylidis et al., 2014; Almeida-García et al., 2016; Lin et al., 2017; Strzelecka et al., 2017), few studies have comprehensively evaluated the relationship among tourism empowerment, residents’ participation willingness and residents’ participation behaviors. Therefore, the second purpose of this study is to analyze the internal mechanism of the influence of tourism empowerment on ethnic village residents’ participation behavior in tourism poverty alleviation from the perspective of villagers’ participation willingness, test the mediating effect of villagers’ participation willingness and theoretically expand the understanding of the impact mechanism of tourism empowerment.

Though previous studies confirmed that tourism empowerment had a positive impact on participation willingness (Liu and Li, 2016), this effect is the same for different village resident individuals needs to be revealed (Fotiadis et al., 2019). In minority areas, the lack of poor peasant households’ ability is the main reason for their low participatory tourism willingness (Wang et al., 2010; Lü, 2012). Yang and Ba (2012) proposed that only through the ways of strengthening the training of tourism knowledge and skills and the training of tourism management mode, Mandarin and other knowledge and abilities can encourage impoverished population participate in tourism poverty alleviation to the greatest extent. Individual characteristics and individual social and cultural factors (such as age, gender, cultural background, experience, personality, etc.) will indirectly affect behavioral attitudes, subjective norms and perceived behavioral control through influencing behavioral beliefs, and ultimately affect behavioral intentions and specific behaviors (Lim and Dubinsky, 2005). Various ability factors such as the health status and education level affect residents’ willingness to participate in tourism poverty alleviation (León, 2007; Lu et al., 2017). The influence of tourism empowerment on villagers’ participation willingness may vary owing to the differences in their participation ability. Therefore, the third aim of this study is to investigate the moderating effect of ethnic village residents’ participation ability in tourism poverty alleviation between tourism empowerment and villagers’ participation willingness.

We hope to contribute to the literature on tourism poverty alleviation and tourism empowerment for ethnic village residents in the following ways. First, by taking tourism empowerment as the antecedent variable of ethnic village residents’ participation behavior in tourism poverty alleviation, to a certain extent, fills the gap in the long absence of empirical research to explore the relationship between the two, and helps explain how to motivate ethnic village residents’ participation behavior in tourism poverty alleviation. At the same time, our research not only helps to enrich the theoretical system of tourism empowerment, but also helps to construct the framework of tourism empowerment in practice. By introducing the power relationship into the study of tourism poverty alleviation in ethnic villages, it provides a new perspective and theoretical breakthrough point for us to explore the ways and effective modes for ethnic village residents to participate in tourism poverty alleviation, which is also a reflection of the accelerating penetration and integration trend of tourism science and other social sciences in recent years. Second, by exploring the mediating effect of residents’ willingness to participate in tourism poverty alleviation in ethnic villages, our study discusses how residents’ willingness to participate in tourism poverty alleviation helps to explain the effectiveness of tourism empowerment, so as to provide new ideas for relevant research on participation willingness. Third, by investigating the moderating effect of poverty alleviation ability of ethnic village residents participating in tourism, our study clarified the potential boundary conditions for the effectiveness of tourism empowerment. In addition, we also hope that this study will help relevant authorities to formulate management countermeasures for stimulating ethnic village residents to participate in tourism poverty alleviation, so as to generate new breakthroughs in tourism poverty alleviation in ethnic village and accelerate the pace of poverty alleviation for ethnic village residents.

The overall structure of this paper is as follows. In section “Theoretical Overview and Research Hypotheses,” the theoretical overview of tourism empowerment, participation behavior, participation willingness, and participation ability is conducted to extract the research hypotheses developed. Section “Materials and Methods” mainly explains the research design and measurement. The analysis results of reliability and validity, correlation, main effect, mediating effect, and moderating effect are shown in section “Results.” Finally, we elaborate on the theoretical contributions, management implications, limitations, and future research.

## Theoretical Overview and Research Hypotheses

### Tourism Empowerment and Participation Behavior

Empowerment is first proposed by Solomon (1976) in the late 1970s, it refers to the process of improving individuals’ and groups’ ability and awareness of rights through external intervention and assistance, thereby reducing or eliminating their sense of powerlessness (Zimmerman, 1990). Sen’s (1981) theory of right poverty argues that empowering the poor is the fundamental approach to solve the problem of poverty. In the field of tourism research, tourism empowerment is the research object that attracts the scholars’ concern (Boley et al., 2018). Akama (1996) firstly proposed the initial function of tourism empowerment. Tourism empowerment means that stimulating the potential advantages of community residents and increasing their influence and control over the internal and external environment when the established rights remain unchanged, to expanding residents’ rights and promoting the transfer and redistribution of residents’ rights and interests under the condition that the democratic consciousness and resource control of community residents are generally improved (Zhang, 2013). Tourism empowerment is a multi-dimensional concept, Scheyvens (1999) proposed that tourism empowerment should be divided into four dimensions: tourism economic empowerment, tourism psychological empowerment, tourism social empowerment, and tourism political empowerment, the four-dimensional framework has been widely recognized by the academic community. Sofield (2003) further specified the concept, theory and method of tourism empowerment.

The essence of poverty alleviation process of tourism in ethnic villages is regarded as the process of each stakeholder’s possession, distribution and use of tourism resources, and the process of rights game and exchange. From the actual situation, the lack of ethnic village tourism resources property rights, and the dual impact of market economy after the formation of the atomization state caused by the high cost of cooperation tends to make the community interact with external stakeholders and game often in a state shall not be entitled to. Therefore, it is necessary to combine the two issues of power in politics with poverty alleviation through tourism.

Ethnic village residents’ behavior to participate in tourism poverty alleviation means that villagers participate in various affairs activities on the process of tourism poverty alleviation, for instance, taking part in song and dance performances, displaying ethnic culture, providing accommodation and catering, selling commodities with ethnic characteristics, and participating in decision-making, management, and supervision of ethnic village tourism. Tourism empowerment can increase the opportunities for residents to participate in tourism poverty alleviation, which in turn has a direct positive impact on residents’ participation behavior (Zhang, 2015). Thus, tourism empowerment may affect the behavior of residents to participate in tourism poverty alleviation.

Firstly, tourism empowerment is the most direct characteristic of tourism, and economic influence is the most obvious characteristic of tourism. Tourism economic empowerment means that the development of tourism significantly improves the employment rate, income, and living standard of community residents (Scheyvens, 1999). For the residents who treat tourism as a means of livelihood (Su et al., 2016), it will become an important direct motivation for them to actively participate in tourism poverty alleviation. Indeed, community tourism does greatly promote the development of local economy, and also solves the way out of part of the surplus labor force. The participation rate of community residents in tourism also increases rapidly.

Secondly, tourism psychological empowerment means that the mental status of residents can be changed in tourism poverty alleviation. If the common rural scenery and traditional ethnic customs have been recognized by non-local tourists, residents’ pride and self-confidence might have been effectively enhanced (Scheyvens, 1999). Stronza and Gordillo (2008) confirmed that the pride of residents in an Amazon community is the most important non-economic benefit in the development of local tourism. Realizing the uniqueness and value of the local community’s traditional culture, natural resources and traditional knowledge, the villagers are proud of their own culture and tradition from the bottom of their hearts and actively participate in poverty alleviation through tourism (Wang, 2013).

Thirdly, according to Maslow’s “hierarchy of needs,” it suggests that harmonious interpersonal communication is one of the basic needs of individuals. Tourism social empowerment shows that the overall sense of community has been confirmed and strengthened by the combination of tourism development and community construction (Sun, 2008). It encourages residents to involve themselves in tourism poverty alleviation for the sake of satisfying their social needs by enhancing the cohesion and cooperation among residents of ethnic villages. Perkins and Zimmerman (2004) illustrated that the empowerment of the tourism community contributes to the improvement of the quality of life of the community and the linkage of community organizations. In the meanwhile, the increasing power of tourism society includes the growth of some groups recognized by all communities, the development of community public services, and the strengthening of the overall image of the community. As for the community, the government and enterprises are the external stakeholders of the tourism industry, and the internal stakeholders of the community also need to mobilize and integrate to form a joint force. On this basis, a new community organization system is built to prepare for the effective participation of the community.

Lastly, the lack of residents’ political rights is the root of the failure of community participation and the main reason for hindering the healthy development of tourism (Zuo and Bao, 2008). Tourism political empowerment emphasizes providing residents with approaches and opportunities to express their opinions and participate in decision-making, management, and supervision of tourism (Scheyvens, 1999), which means, in this way, it can encourage residents to have the inner motivation to achieve real participation by giving them full decision-making and discourse power. If the local government does not provide relevant approaches for residents, the possibility of residents participating in tourism development will be greatly reduced (Aas et al., 2005). Given the above analysis, we propose the following hypothesis:

Hypothesis 1a (H1a): Tourism economic empowerment positively affects ethnic village residents’ behavior participating in tourism poverty alleviation.

Hypothesis 1b (H1b): Tourism psychological empowerment positively affects ethnic village residents’ behavior participating in tourism poverty alleviation.

Hypothesis 1c (H1c): Tourism social empowerment positively affects ethnic village residents’ behavior participating in tourism poverty alleviation.

Hypothesis 1d (H1d): Tourism political empowerment positively affects ethnic village residents’ behavior participating in tourism poverty alleviation.

### The Mediating Role of Participation Willingness

Ethnic village residents’ willingness to participate in tourism poverty alleviation refers to the subjective desire of villagers to participate in tourism poverty alleviation, including the desire to engage in tourism-related work, tourism-related activities, and tourism development affairs of ethnic village. Social exchange theory states that residents as rational people, the formation of their participation willingness is based on the principle of reciprocity and mutual benefit, the premise to stimulate their participation willingness is that their perceived benefits are greater than perceived costs (Vargas-Sanchez et al., 2011). When residents’ comprehensive income from tourism development exceeds their expectations, they will have a positive participation attitude (Yang et al., 2005).

Specifically, tourism economic empowerment effectively protects the tourism economic incomes of residents by establishing a reasonable benefit distribution mechanism. Keogh (1990) showed that there is a significant positive correlation between residents’ perception of the impact on tourism economic interests and their attitude toward tourism support. The greater the economic interests of tourism development perceived by residents, the more inclined they are to support the development of tourism. Current studies have proved that residents’ attitudes toward tourism were positively correlated with their tourism economic benefits (López et al., 2018). The more benefits residents gain, the more active participation attitude toward tourism development they hold (Ouyang et al., 2017). The most direct influence of tourism psychological empowerment on residents is leading them to form the consciousness of national culture (Di Castri, 2004). Boley et al. (2014) took three villages in the United States as research objects, the empirical results found that psychological empowerment promoted residents’ support for tourism development by inspiring their self-confidence. Tourism social empowerment means that the tourism activities undertaken by residents promote the internal balance of the village and standardization of the social order. The research conducted by Liu and Li (2016) based on the ethnic villages of Kanas community in Xinjiang shows that social empowerment can boost the development of the community and positively affect residents’ support for tourism. The realization of tourism political empowerment offers villagers various approaches to express their demands and appeals for building up a sound extension mechanism. It affects residents’ willingness to participate in tourism by influencing their trust toward local government actors (Nunkoo and Ramkissoon, 2012).

We believe that the theory of planned behavior can effectively analyze the behavioral characteristics of ethnic village residents participating in tourism poverty alleviation. According to the theory of planned behavior, the participation willingness of ethnic village residents is the inherent reason and direct motivation to drive their behavior of participating in tourism poverty alleviation. The stronger is the participation willingness of village residents, the more likely they participate in tourism poverty alleviation. As an indicator to measure residents’ acceptability for developing tourism (Andriotis, 2005), participation willingness can predict residents’ participation behavior. Most studies have proved that there is a significant correlation between behavioral intention and actual behavior (Zeihaml et al., 1996). Besides, Huang et al. (2014) and Lu et al. (2017) empirically studied the relationship between residents’ willingness and behavior to participate in tourism development, the findings suggested that the former one had a significant positive effect on the latter one. Based on the above discussion, we put forward the following hypotheses:

Hypothesis 2a (H2a): Participation willingness plays a mediating role between tourism economic empowerment and ethnic village residents’ behavior participating in tourism poverty alleviation.

Hypothesis 2b (H2b): Participation willingness plays a mediating role between tourism psychological empowerment and ethnic village residents’ behavior participating in tourism poverty alleviation.

Hypothesis 2c (H2c): Participation willingness plays a mediating role between tourism social empowerment and ethnic village residents’ behavior participating in tourism poverty alleviation.

Hypothesis 2d (H2d): Participation willingness plays a mediating role between tourism political empowerment and ethnic village residents’ behavior participating in tourism poverty alleviation.

### The Moderating Role of Participation Ability

Sen’s (1999) theory of feasible ability holds that the welfare of the individual is secured by individual ability, the cause of poverty is lack of ability, which is composed of a series of functions, such as the function of avoiding hunger, disease, and receiving education. Banerjee and Duflo (2011) further point out that one of the reasons why poor people are unable to get rid of poverty is that their ability to obtain the right information is limited, instead, they would like to believe in the unreal information. They also argue that poor people might have the weak self-cognition ability and seem to give up the long-term planning to choose the short-sightedness and stereotypes (Hung et al., 2011).

Ethnic village residents’ ability to participate in tourism poverty alleviation refers to the subjective conditions possessed by residents and conducive to their participation in tourism poverty alleviation, such as relevant tourism basic knowledge and skills, abundant personal time and certain economic resources, and information acquisition capabilities (Hung et al., 2011). The participation behavior of poor farmers is characterized by significant ability dependence, those with low participation ability have the problem of insufficient participation (Tian and Zhang, 2018). The willingness of poor people to participate in tourism poverty alleviation varies significantly among poor people with different population characteristics (Lu et al., 2017). The study of Wang and Xiang (2017) found that community residents’ participation ability had a significant positive impact on their participation motivation. This is in accordance with the view held by Li and Liu (2019), their research indicated that if community residents could fully understand tourism poverty alleviation policies and possess relevant skills, their enthusiasm for participating in tourism poverty alleviation would increase.

Past studies usually measured the level of individuals’ participation ability according to their self-perception of whether they could perform a specific behavior (Bandura, 1977). Residents with high participation ability are more likely to recognize the convenient conditions, and they have strong self-confidence when facing challenges. Nevertheless, residents with low participation ability perceive more barriers to implementation, they have lower self-confidence and tend to be more anxious. Ethnic village residents have different abilities to participate in tourism, their participation intentions also differ significantly (Li et al., 2009). For residents with high participation ability, they are more likely to recognize their own advantages in the process of tourism poverty alleviation, and believe that participating in tourism poverty alleviation is an important opportunity for them to get rid of poverty and become prosperous (Sun, 2008). There are inter-positive relations among tourism empowerment, recognition, and willingness, that is, the greater the benefits brought by tourism empowerment, the higher the residents’ recognition of tourism poverty alleviation, and the stronger their willingness to participate in tourism poverty alleviation. On the contrary, residents with low participation ability lack confidence in their capacity to perform relevant work of tourism poverty alleviation activities. Even if tourism empowerment is fully realized, their participation willingness will not be actually strong (Yang, 2017). To summarize, we propose the following hypotheses:

Hypothesis 3a (H3a): Participation ability positively moderates the relationship between tourism economic empowerment and ethnic village residents’ willingness participating in tourism poverty alleviation.

Hypothesis 3b (H3b): Participation ability positively moderates the relationship between tourism psychological empowerment and ethnic village residents’ willingness participating in tourism poverty alleviation.

Hypothesis 3c (H3c): Participation ability positively moderates the relationship between tourism social empowerment and ethnic village residents’ willingness participating in tourism poverty alleviation.

Hypothesis 3d (H3d): Participation ability positively moderates the relationship between tourism political empowerment and ethnic village residents’ willingness participating in tourism poverty alleviation.

**FIGURE 1:**
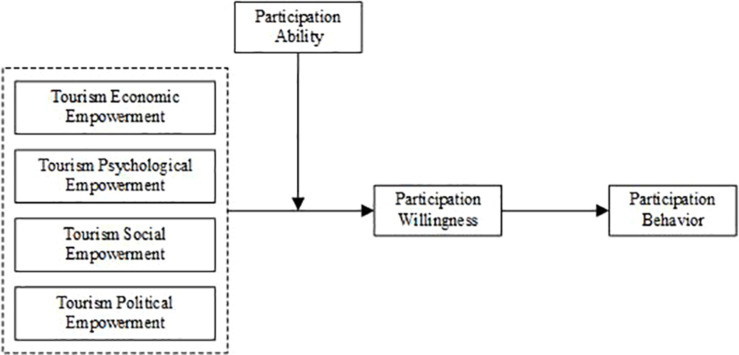
Theoretical model.

Based on the above theoretical views and research hypotheses, the theoretical model of this study is shown in Figure 1.

## Materials and Methods

### Study Area

Zenlei Village, which is one of the first batches of traditional villages in China, is in Dujiang Town, Sandu Shui Autonomous County, Guizhou Province. It lies in the mountains of the watershed between the upper reaches of the Duliujiang River and the Longjiang River. The history of this village is more than 300 years ago, and it is a well-known Chinese historical and cultural village with its original ecological national culture being well-protected. According to the data provided by the village committee in August 2019, there are 236 peasant households with a population of 1054 in Zenlei Village, of which 65% are Shui nationality, mainly living in Shangzhai and Xiazhai, 35% are Miao nationality, mainly living in Zhongzhai and Paichangzhai. The Shui and Miao compatriots live in harmony and depend on each other for mutual development. Zenlei Village has a beautiful natural environment and is a tourist resort integrating natural ecology, cultural landscapes, and folk customs. It has been chosen as one of the famous Chinese tourist villages with cultural traits. Since the village began to develop tourism early this century, it has gotten a sound economic and social benefit and had a profound influence on the villagers’ labor life. In recent years, Zenlei Village has positively responded to the national strategy of “targeted poverty alleviation, industrial poverty alleviation,” encouraging village residents to vigorously develop ethnic cultural tourism and actively participate in local tourism poverty alleviation, and striving to motivate them to achieve both material and mental poverty alleviation. At present, Zenlei Village has achieved staged aims, forming a characteristic poverty alleviation development mode of “courtyard economy plus mountain tourism.” Thus, it is reasonable to choose Zenlei Village as a case to study the tourism poverty alleviation of ethnic villages, as it is typical and representative.

### Sample and Procedures

To reduce the impact of common method variance, the research group conducted two surveys. From June 20 to 29, 2018, the research group conducted a quantitative study in Zenlei Village by distributing the questionnaires, and made a supplementary survey in the village on August 9 to 11, 2019. The participants in this study were the residents of Zenlei Village. They filled out questionnaires depending on their actual situation. For the residents who were illiterate or semiliterate, the substitute-filling questionnaire method was applied. All questionnaires were collected by the research group on the spot. The data reflected the subjective judgment of residents.

A total of 248 residents participated in the questionnaire survey. After rejecting the invalid questionnaires, 239 valid questionnaires were finally retained. The effective recovery rate was 96.37%. The results obtained from the descriptive statistics analysis are presented in Table 1. It can be seen from Table 1 that there was a significant difference between the males and females ratio, with males accounting for 60.25% and females accounting for 39.75%. Most of the respondents aged between 30 and 39, accounting for 25.94%. The educational level of respondents was mainly in primary school and never attended school, accounting for 41.84% and 31.38% respectively. The per capita annual income of respondents was mainly distributed between 3001 and 5000 yuan, accounting for 29.29%. The residence length of respondents was mainly distributed in 21 years and above, accounting for 69.87%.

**TABLE 1:** Descriptive statistics.

**Characteristic**	**Category**	**Frequency**	**Percentage**
Gender	Male	144	60.25
	Female	95	39.75
Age	20 and below	19	7.95
	21 to 29	35	14.64
	30 to 39	62	25.94
	40 to 49	48	20.08
	50 to 59	40	16.74
	60 and above	35	14.64
Education level	Never attended school	75	31.38
	Primary school	100	41.84
	Middle school	51	21.34
	High school and above	13	5.44
Per capita annual income	Less than 2300 yuan	44	18.41
	2301 to 3000 yuan	57	23.85
	3001 to 5000 yuan	70	29.29
	5001 to 8000 yuan	31	12.97
	8001 to 11000 yuan	15	6.28
	11001 yuan and above	22	9.21
Residence length	5 years and below	12	5.02
	6 to 10 years	39	16.32
	11 to 20 years	21	8.79
	21 years and above	167	69.87

### Measures

This study used the existing domestic maturity scales as the measurement tool. Except for controlled variables, all other variables were scored with a five-point Likert scale ranging from 1 (strongly disagree) to 5 (strongly disagree). The measurement of independent variable tourism empowerment adopted the scale developed by Wang et al. (2015), including four dimensions: tourism economic empowerment, tourism psychological empowerment, tourism social empowerment, and tourism political empowerment, with a total of 13 items. Among them, tourism economic empowerment includes three items, one example is “Part of my income comes from the development of local tourism,” the reliability of this scale is 0.81. Tourism psychological empowerment includes three items, one example is “I am proud of being a villager in this village,” the reliability of this scale is 0.84. Tourism social empowerment includes three items, one example is “The development of tourism deepens my relationship with others in this village,” the reliability of this scale is 0.81. Tourism political empowerment includes four items, one example is “I can participate in the formulation of local tourism development plans,” the reliability of this scale is 0.84. The scale in the study of Yang et al. (2005) which includes six items was used to measure the dependent variable participation behavior, an example item is “I often participate in the establishment of village tourism policies,” and the reliability of this scale is 0.86. The mediator variable participation willingness was measured by using a scale which is developed by Huang (2012) and contains seven items, an example item is “I support the development of local tourism,” and the reliability of this scale is 0.88. The moderator variable participation ability was measured by using a scale from Yang et al. (2005) that contains six items in total, an example item is “Our family has certain economic conditions, and we can provide certain financial assistance for tourism poverty alleviation,” and the reliability of this scale is 0.83. Additionally, demographic characteristic variables including gender, age, educational level, per capita annual income, and residence length were used as controlled variables.

## Results

### Reliability and Validity Analysis

We used Amos 21.0 and SPSS 21.0 for reliability and validity analysis. As shown in Table 2, the Cronbach’s alpha (CA) and composite reliability (CR) of all latent variables are greater than 0.70, indicating that the measurement model has expected reliability. The average variance extracted (AVE) of each latent variable is greater than or very close to 0.50, showing that the convergent validity of the measurement model is sound. It can be seen from Table 3 that the square root of AVE of each latent variable is greater than its correlation coefficient with other latent variables, reflecting that the measurement model has good discriminant validity.

**TABLE 2:** Results of reliability and validity analysis.

**Latent variable**	**Measurement item**	**Factor loading**	**AVE**	**CR**	**CA**
Tourism economic empowerment (ECE)	ECE1	0.83***	0.62	0.83	0.81
	ECE2	0.90***			
	ECE3	0.60***			
Tourism psychological empowerment (PSE)	PSE1	0.78***	0.63	0.84	0.84
	PSE2	0.77***			
	PSE3	0.83***			
Tourism social empowerment (SOE)	SOE1	0.81***	0.59	0.81	0.81
	SOE2	0.84***			
	SOE3	0.64***			
Tourism political empowerment (POE)	POE1	0.77***	0.54	0.82	0.84
	POE2	0.74***			
	POE3	0.70***			
	POE4	0.72***			
Participation willingness (PAW)	PAW1	0.47***	0.53	0.88	0.88
	PAW2	0.64***			
	PAW3	0.72***			
	PAW4	0.80***			
	PAW5	0.82***			
	PAW6	0.83***			
	PAW7	0.73***			
Participation ability (PAA)	PAA1	0.68***	0.44	0.83	0.83
	PAA2	0.59***			
	PAA3	0.70***			
	PAA4	0.72***			
	PAA5	0.67***			
	PAA6	0.65***			
Participation behavior (PAB)	PAB1	0.68***	0.52	0.86	0.86
	PAB2	0.64***			
	PAB3	0.53***			
	PAB4	0.78***			
	PAB5	0.87***			
	PAB6	0.76***			

***p < 0.001.

**TABLE 3:** Results of correlation analysis.

**Variable**	**1**	**2**	**3**	**4**	**5**	**6**	**7**	**8**	**9**	**10**	**11**	**12**
(1) Gender	–											
(2) Age	0.14*	–										
(3) Education	0.01	−0.52**	−									
(4) Income	0.04	0.00	0.12	–								
(5) Length	0.16*	0.18**	−0.13*	0.11	–							
(6) ECE	–0.04	0.13	–0.05	0.20**	0.14**	**0.79**						
(7) PSE	0.11	0.09	0.02	0.17**	0.21**	0.38**	**0.79**					
(8) SOE	0.11	0.01	0.03	0.20**	0.03	0.56**	0.66**	**0.77**				
(9) POE	0.10	0.04	0.10	0.27**	0.14*	0.49**	0.31**	0.48**	**0.73**			
(10) PAW	0.11	0.09	0.13	0.23**	0.10	0.27**	0.53**	0.49**	0.32**	**0.73**		
(11) PAA	0.10	0.04	0.08	0.19**	–0.01	0.24**	0.33**	0.34**	0.34**	0.23**	**0.67**	
(12) PAB	–0.02	0.01	0.11	0.26**	0.00	0.48**	0.37**	0.39**	0.62**	0.36**	0.40**	**0.72**
Mean	0.60	3.67	2.01	2.92	3.44	2.68	3.66	3.20	2.41	3.79	3.08	2.37
*SD*	0.49	1.49	0.87	1.48	0.94	1.12	1.00	1.09	1.08	0.94	0.95	0.95

*p < 0.05,**p < 0.01. The bold value on the diagonal is the square root of AVE.

### Correlation Analysis

Table 3 shows the mean, standard deviation, and correlation coefficient of each variable. From Table 3 we can see that tourism economic empowerment (*r* = 0.48, *p* < 0.01), tourism psychological empowerment (*r* = 0.37, *p* < 0.01), tourism social empowerment (*r* = 0.39, *p* < 0.01), and tourism political empowerment (*r* = 0.62, *p* < 0.01) are significantly and positively correlated with participation behavior. Also, tourism economic empowerment (*r* = 0.27, *p* < 0.01), tourism psychological empowerment (*r* = 0.53, *p* < 0.01), tourism social empowerment (*r* = 0.49, *p* < 0.01), and tourism political empowerment (*r* = 0.32, *p* < 0.01) are also significantly and positively related to participation willingness. Hypotheses H1a, H1b, H1c, and H1d are preliminarily supported. Moreover, participation willingness has obviously positive correlation with participation behavior (*r* = 0.36, *p* < 0.01). Hypotheses H2a, H2b, H2c, and H2d receive preliminarily support. Furthermore, participation ability is significantly and positively related to tourism economic empowerment (*r* = 0.24, *p* < 0.01), tourism psychological empowerment (*r* = 0.33, *p* < 0.01), tourism social empowerment (*r* = 0.34, *p* < 0.01), tourism political empowerment (*r* = 0.34, *p* < 0.01), and participation willingness (*r* = 0.23, *p* < 0.01). Hypotheses H3a, H3b, H3c, and H3d are preliminarily supported. According to the above results, the relevant hypotheses of this study have been initially validated.

### Analysis of the Main Effect and Mediating Effect

According to the steps proposed by Baron and Kenny (1986), we examined whether participation willingness plays a mediating effect between tourism empowerment and participation behavior. The analysis results are shown in Table 4. First, taking participation willingness as the dependent variable, controlled variables (gender, age, education level, per capita annual income, and residence length) are included in the regression equation to build the model M1. On the basis of the model M1, independent variables (tourism economic empowerment, tourism psychological empowerment, tourism social empowerment, and tourism political empowerment) are successively entered the regression equation to build the models M2, M3, M4, and M5, so as to test the relationships between tourism economic empowerment, tourism psychological empowerment, tourism social empowerment, tourism political empowerment, and participation willingness. From the models M2, M3, M4, and M5, we can see that tourism economic empowerment (M2, β = 0.23, *p* < 0.001), tourism psychological empowerment (M3, β = 0.49, *p* < 0.001), tourism social empowerment (M4, β = 0.46, *p* < 0.001), and tourism political empowerment (M5, β = 0.25, *p* < 0.001) have significant positive effects on participation willingness.

**TABLE 4:** Analysis results of the main effect and mediating effect.

**Variable**	**Participation willingness**					**Participation behavior**									
	
	**M1**	**M2**	**M3**	**M4**	**M5**	**M6**	**M7**	**M8**	**M9**	**M10**	**M11**	**M12**	**M13**	**M14**	**M15**
Gender	0.06	0.08	0.03	0.01	0.05	–0.04	0.00	–0.06	–0.08	–0.74	–0.06	–0.02	–0.07	–0.08	–0.08
Age	0.17	0.14	0.12	0.17	0.15	0.09	0.03	0.05	0.09	0.03	0.03	0.00	0.03	0.06	0.01
Education	0.20	0.20	0.16	0.20	0.17	0.12	0.11	0.09	0.12	0.04	0.06	0.07	0.06	0.08	0.01
Income	0.20	0.15	0.13	0.11	0.14	0.25	0.16	0.20	0.18	0.10	0.19	0.13	0.18	0.16	0.08
Length	0.07	0.04	–0.02	0.07	0.04	–0.02	–0.07	–0.08	–0.02	–0.08	–0.04	–0.08	–0.08	–0.03	–0.09
ECE		0.23***					0.46***					0.41***			
PSE			0.49***					0.35***					0.26***		
SOE				0.46***					0.36***					0.28***	
POE					0.25***					0.61***					0.57***
PAW											0.31***	0.22***	0.18*	0.18*	0.17**
*R*^2^	0.10	0.15	0.32	0.30	0.15	0.08	0.27	0.19	0.20	0.41	0.17	0.31	0.21	0.22	0.44
Δ*R*^2^	—	0.05	0.23	0.20	0.06	—	0.19	0.11	0.12	0.33	0.09	0.23	0.14	0.15	0.36
*F*	5.07	6.64***	18.46***	16.35***	7.04***	3.96	14.37***	9.13***	9.65***	27.01***	7.74***	14.92***	8.95***	9.45***	25.56***

Mi (i = 1, 2,3…) represents model i (i = 1, 2, 3…). *p < 0.05, **p < 0.01, ***p < 0.001.

Second, taking participation behavior as the dependent variable, controlled variables (gender, age, education level, per capita annual income, and residence length) are entered the regression equation to build the model M6. On the basis of the model M6, adding independent variables (tourism economic empowerment, tourism psychological empowerment, tourism social empowerment, and tourism political empowerment) in turn to construct the models M7, M8, M9, and M10, in order to examine the relationships between tourism economic empowerment, tourism psychological empowerment, tourism social empowerment, tourism political empowerment, and participation behavior. From the models M7, M8, M9, and M10, it refers that tourism economic empowerment (M7, β = 0.46, *p* < 0.001), tourism psychological empowerment (M8, β = 0.35, p < 0.001), tourism social empowerment (M9, β = 0.36, *p* < 0.001), and tourism political empowerment (M10, β = 0.61, *p* < 0.001) have significant positive effects on participation behavior. Hypotheses H1a, H1b, H1c, and H1d are further supported. Third, using participation behavior as the dependent variable, controlled variables (gender, age, education level, per capita annual income, and residence length) and mediating variable (participation willingness) are simultaneously incorporated into the regression equation to build the model M11, and test the relationship between participation willingness and participation behavior. According to the model M11, participation willingness has a significant positive influence on participation behavior (M11, β = 0.31, *p* < 0.001).

Finally, using participation behavior as the dependent variable, controlled variables (gender, age, education level, per capita annual income, and residence length), independent variables (tourism economic empowerment, tourism psychological empowerment, tourism social empowerment, and tourism political empowerment), and mediating variable (participation willingness) are simultaneously entered the regression equation to build the models M12, M13, M14, and M15, the aim is testing the mediating role of participation willingness in the relationships between tourism economic empowerment, tourism psychological empowerment, tourism social empowerment, tourism political empowerment, and participation behavior. From the model M12, it shows participation willingness still has a significant positive effect on participation behavior (β = 0.22, *p* < 0.001), while the influence of tourism economic empowerment on participation behavior is weakened (β = 0.41, *p* < 0.001), yet still maintains a remarkable level, this illustrates that participation willingness partially mediates the influence of tourism economic empowerment on participation behavior. Hypothesis H2a is further supported. Similarly, it can be concluded from the models M13, M14, and M15 that participation willingness plays a partial mediating role in the relationships between tourism psychological empowerment, tourism social empowerment, tourism political empowerment, and participation behavior. Hypotheses H2b, H2c, and H2d are further supported.

### Regulating Effect Analysis

This study used hierarchical regression to test whether participation ability moderates the relationship between tourism empowerment and participation willingness. The specific results are shown in Table 5. In order to minimize the problem of multicollinearity among variables, independent variables, and moderating variable are respectively centralized before constructing the interaction terms of independent variables (tourism economic empowerment, tourism psychological empowerment, tourism social empowerment, and tourism political empowerment) and moderating variable (participation ability). Taking participation willingness as the dependent variable, controlled variables (gender, age, education level, per capita annual income, and residence length), independent variables (tourism economic empowerment, tourism psychological empowerment, tourism social empowerment, and tourism political empowerment), moderating variable (participation ability) and the interaction terms of independent variables and moderating variable are simultaneously included in the regression equation. The models M20, M21, M22, and M23 are constructed to test the moderating effect of participation ability on the relationships between tourism economic empowerment, tourism psychological empowerment, tourism social empowerment, tourism political empowerment, and participation willingness. From the models M20 and M21, the interaction terms of tourism economic empowerment (M20, β = 0.14, *p* < 0.05), tourism psychological empowerment (M21, β = 0.13, *p* < 0.05), and participation ability have significant positive effects on participation willingness, it suggests that participation ability can positively moderate the relationships between tourism economic empowerment, tourism psychological empowerment, and participation willingness. Hypotheses H3a and H3b are further supported. However, from the models M22 and M23, it can be seen that the interaction items of tourism social empowerment (M22, β = 0.08, *p* > 0.05), tourism political empowerment (M23, β = 0.11, *p* > 0.05), and participation ability has no significant influence on participation willingness, which indicates that participation ability has no moderating effect on the relationships between tourism social empowerment, tourism political empowerment, and participation willingness. Hypotheses H3c and H3d are not supported.

**TABLE 5:** Analysis results of regulating effect.

**Variable**	**Participation willingness**							
	**M16**	**M17**	**M18**	**M19**	**M20**	**M21**	**M22**	**M23**
Gender	0.07	0.03	0.01	0.04	0.05	0.02	0.01	0.03
Age	0.13	0.12	0.17	0.14	0.13	0.10	0.15	0.12
Education	0.18	0.16	0.20	0.16	0.17	0.15	0.19	0.15
Income	0.14	0.13	0.10	0.13	0.14	0.11	0.10	0.12
Length	0.05	–0.02	0.07	0.05	0.04	–0.03	0.07	0.05
ECE	0.20**				0.19**			
PSE		0.49***				0.49***		
SOE			0.45***				0.44***	
POE				0.22**				0.19**
PAA	0.13	0.02	0.03	0.11	0.19**	0.06	0.06	0.14*
ECE × PAA					0.14*			
PSE × PAA						0.13*		
SOE × PAA							0.08	
POE × PAA								0.11
*R*^2^	0.16	0.32	0.30	0.16	0.18	0.34	0.30	0.17
Δ*R*^2^	0.06	0.23	0.20	0.07	0.08	0.24	0.21	0.08
*F*	6.31***	15.78***	14.00***	6.46***	6.17***	14.66***	12.49***	6.03***

Mi (i = 1, 2, 3…) represents model i (i = 1, 2, 3…). *p < 0.05, **p < 0.01, ***p < 0.001.

**FIGURE 2:**
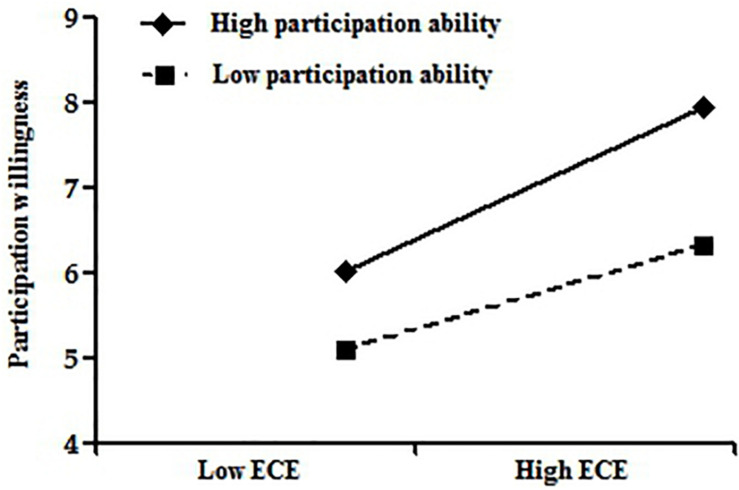
Moderating effect of participation ability on the relationship between tourism economic empowerment and participation willingness.

**FIGURE 3:**
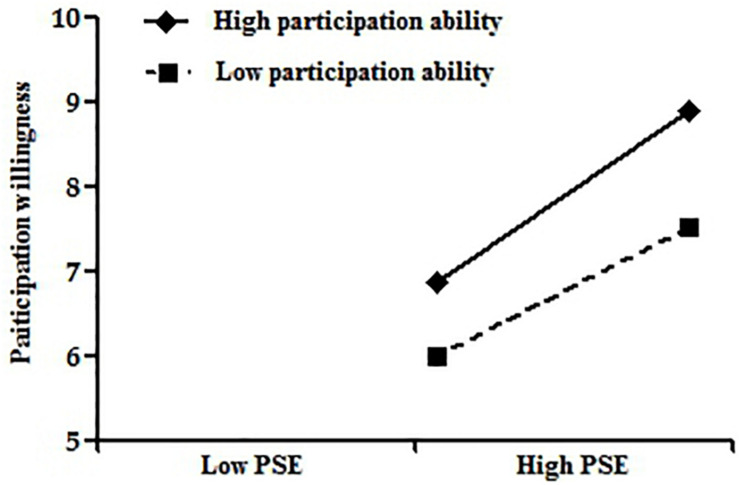
Moderating effect of participation ability on the relationship between tourism psychological empowerment and participation willingness.

For further and intuitive observation of the moderating effect of participation ability on the relationships between tourism economic empowerment, tourism psychological empowerment, and participation willingness, we plotted the moderating effect chart according to a standard deviation above or below the mean of participation ability according to the method recommended by Aiken and West (1991). As can be seen from Figure 2, there is a strong positive correlation between tourism economic empowerment and participation willingness when residents’ participation ability is strong. The positive correlation between tourism economic empowerment and participation willingness is weak when residents’ participation ability is low. It can be seen from Figure 3 that there is a strong positive correlation between tourism psychological empowerment and participation willingness when residents’ participation ability is high. The positive correlation between tourism psychological empowerment and participation willingness is weak when residents’ participation ability is low.

## Discussion

In this study, the ability of ethnic village residents to participate in tourism poverty alleviation was introduced as a moderator variable, and a structural relationship model was established among tourism empowerment, residents’ willingness to participate in tourism poverty alleviation and residents’ participation behavior in tourism poverty alleviation. Through the reliability and validity analysis and correlation analysis of the questionnaire data, and the test of the basic hypothesis of this study, and then to explore the factors affecting the participation behavior of ethnic village residents in tourism poverty alleviation, the following three conclusions are drawn.

First, tourism empowerment has a significant impact on the formation of tourism poverty alleviation activities for ethnic village residents. This conclusion further expands the research results of Lü (2012). Second, the willingness of ethnic village residents to participate in tourism poverty alleviation plays a part of the mediating role in the relationship between tourism empowerment and residents’ participation behavior in tourism poverty alleviation. The conclusion of this study complements the existing studies (Stylidis et al., 2014; Almeida-García et al., 2016; Lin et al., 2017; Strzelecka et al., 2017). Thirdly, the ability of ethnic village residents to participate in tourism poverty alleviation positively regulates the relationship between tourism economic empowerment, tourism psychological empowerment and the residents’ willingness to participate in tourism poverty alleviation. However, the ability of ethnic village residents to participate in tourism poverty alleviation does not play a moderating role between tourism social empowerment, tourism political empowerment and participation willingness.

In terms of tourism social empowerment, residents in ethnic villages compete for tourism development benefits in tourism activities, and residents with low participation ability may have negative emotions such as jealousy and hatred, thus reducing their perception of tourism social empowerment. However, in ethnic villages, in addition to grassroots organizations such as village branch and village committee, there are also traditional community organizations such as village old organization (villagers call them “old people”) (Yang, 2017). As representatives of the villagers’ interests, the old village organizations will intervene in the disorderly competition. For example, residents of Zenlei Village used to sell various handicrafts to customers and quarrel with each other for competing for customers, which not only damaged the image and reputation of the ethnic village, but also hurt the feelings between residents of the village. Faced with this chaotic situation, the old organization of the village called a meeting of the villagers, and agreed that on the circle of the village collective performance venue, all the sales booths were uniformly designated number, a family, a fixed number, drawing to decide the seller of each handicraft. Such rules can restore the order of the village in time and effectively, but will not reduce the perception of power increase in tourism society of the residents with low participation ability. Therefore, the ability of ethnic village residents to participate in tourism poverty alleviation does not play a moderating role between tourism social empowerment and participation willingness.

Regarding to tourism political empowerment, as a result of ethnic village tourism poverty alleviation is a kind of “top–down” government behavior, at all levels of government organization attaches great importance to the impoverished residents of low participation ability, to each of the poor by inputting tent card, in-depth analysis of poverty causes, using “target support” “one to one support measures” help the poor residents whose participation ability is low. In the process of assisting, governments at all levels should first understand the basic demands of poor residents with low participation ability, and feed back to the higher authorities to ensure their political rights in the process of poverty alleviation through tourism. Therefore, the ability of ethnic village residents to participate in tourism poverty alleviation does not play a moderating role between tourism political empowerment and willingness to participate.

### Theoretical Contributions

The theoretical contributions of this paper are mainly showing in the following three aspects. First, we demonstrated that tourism empowerment positively affects ethnic village residents’ behavior to participate in tourism poverty alleviation. Tourism empowerment promotes residents to genuinely participate in tourism poverty alleviation by not only increasing residents’ income but creating employment, not only enhancing residents’ self-confidence and pride, but promoting the coordinated development of ethnic village community governance, and ensuring residents’ opportunities to participate in tourism development affairs. At present, empowerment theory is mainly used in the field of sociology, pedagogy, and anthropology, its application in the tourism field is still in the initial stage, so it seems that there is extraordinarily necessary to conduct in-depth research on it (Wang, 2013). Furthermore, former literature discussed the effect of ecotourism cognition (Huang et al., 2014), perceived justice (Xu et al., 2015), participation opportunities (Zhang, 2015), individual characteristics (Simon, 2016), and attitudes to tourism impacts (Ribeiro et al., 2017) on residents’ participatory tourism behavior. Nevertheless, fewer scholars paid attention to the relationship between tourism empowerment and residents’ participatory tourism behavior. We designed tourism empowerment as the independent variable and empirically examined the influence of tourism economic empowerment, tourism psychological empowerment, tourism social empowerment, and tourism political empowerment on participation behavior, which further expanded the research on the driving factors of participation behavior and supplementing related research on tourism empowerment.

Second, we found that participation willingness partially mediates the relationship between tourism empowerment and ethnic village residents’ behavior to participate in tourism poverty alleviation. When the benefits that tourism empowerment brings for residents are higher than the costs that residents pay to participate in tourism poverty alleviation, this will help stimulate residents’ willingness to participate in tourism poverty alleviation, and then enhance their participation behavior. Previous studies examined the relationship between tourism empowerment and participation willingness (Liu and Li, 2016), few of them also explored the effect of participation willingness on participation behavior (Simon, 2016), while fewer literature integrated these three variables into the same framework to study. Taking participation willingness as mediating variable, we uncovered the “black box” of the role of tourism empowerment in participation behavior and analyzed the transmission mechanism of tourism empowerment on participation behavior through participation willingness, this has further enriched the research on the influence mechanism of tourism empowerment and the mediating mechanism between tourism empowerment and participation behavior.

Third, we proved that participation ability plays a positive regulating role in the relationships between tourism economic empowerment, tourism psychological empowerment, and participation willingness. However, it would not affect the relationships between tourism social empowerment, tourism political empowerment, and participation willingness. When residents’ participation ability is high, they would get positive subjective feelings. The higher the economic or mental benefits they gain, the higher their enthusiasm to participate in tourism poverty alleviation. Although current studies have verified that tourism empowerment has a positive effect on residents satisfaction (Ma, 2014), support for tourism (Boley et al., 2014), residents fairness perception (Liu and Li, 2016), and community resilience (Guo et al., 2018), there is a lack of further discussion on the boundary conditions for the effectiveness of tourism empowerment. In this paper, we regarded participation ability as a moderating variable, revealing how participation ability impacts the relationship between tourism empowerment and participation willingness, especially clarifying the function boundary of tourism economic empowerment and tourism psychological empowerment on participation willingness.

### Management Implications

The conclusions obtained in this study provide a basis for inspiring ethnic village residents to participate in tourism poverty alleviation and improving the quality and effectiveness of tourism poverty alleviation. They have vital implications for the management practice of tourism poverty alleviation in ethnic villages, which is manifested in the following three aspects.

First of all, if tourism empowerment has a positive significant influence on residents’ behavior to participate in tourism poverty alleviation, ethnic villages can take the following measures to boost residents’ participation behavior: (a) We should further improve the construction of tourism infrastructure, enrich the content of tourism products, strengthen the development of tourism market, improve the quality of tourism services, and localize the tourism industry chain as much as possible, so as to extend the tourists’ traveling, increase the amount of tourism consumption and expand tourism revenue. We will establish projects to help alleviate poverty through investment and joint venture and cooperation, implement the policy of investing in land management rights and housing property rights, and open up new channels for residents to increase their incomes.

(b) Adequately exploiting, developing, and protecting natural, historical, and cultural resources; developing ethnic cultural tourism with regional characteristics; allowing residents to participate in the performances and activities to display traditional culture, so as to improve their self-confidence of the local culture and enhance the consciousness of inheriting intangible cultural heritage. Attention should be paid to the inter-generational transmission of traditional ethnic culture in villages, and young people should be encouraged to learn their own culture and traditional art, so as to achieve the goal of cultural inheritance.

(c) Social organizations such as tourism associations and tourism coordination groups of tourism chambers of commerce should be established in ethnic villages to increase the social capital of tourism participation of community residents and improve the degree of organization of villagers, so as to reduce the cost of community governance in ethnic villages and improve the capacity building of community participation. Establish village tourism cooperatives, develop village collective economy, and strengthen the residents’ collectivism concept.

(d) Improving stable strategies to participate in tourism decision-making, in order to safeguard the rights of residents, enhance residents’ awareness of their rights, and make residents realize their important roles in tourism development. In formulating relevant tourism policies, the government should reflect the will of the community and widely listen to the opinions and suggestions of villagers. Give the villagers or their representatives an opportunity to state their views before deciding on a community tourism development plan. In the process of tourism development, community residents have channels to express their demands. In the process of project construction, timely inform the villagers of the situation, so that the villagers know the progress of tourism development.

In the next place, tourism empowerment affects residents’ behavior to participate in tourism poverty alleviation through the partial mediating role of participation willingness. This informs that ethnic village can pay special attention to whether tourism empowerment generates residents’ participation willingness. If residents have a positive willingness toward participating in tourism poverty alleviation, tourism empowerment might effectively motivate their participation behavior. Due to the relatively closed ethnic villages, local residents have little understanding of poverty alleviation through tourism, and do not realize the role of tourism in poverty alleviation, so they have a weak sense of participation. Relevant competent departments can deeply understand residents’ willingness of participating in local tourism poverty alleviation through in-depth interviews, complaint cases, and network public opinions. Moreover, they also can increase residents’ participation willingness by strengthening publicity, explaining local tourism poverty alleviation plans, and introducing successful cases and experiences, aiming to reinforce residents’ cognition of tourism poverty alleviation.

On this basis, ethnic villages need to build a reasonable mechanism to turn villagers’ strong desire to participate in tourism poverty alleviation into positive behaviors, so as to ensure villagers’ passionate participation in tourism poverty alleviation. For example, a benefit-sharing mechanism should be established to effectively protect the tourism income of village residents. The construction of the resource compensation mechanism, that is, any land expropriation, land reversal, resource utilization and other actions, must obtain the consent of village residents according to law and regulations, and can only be implemented after the relevant compensation agreement is signed through consultation. We will build a democratic decision-making mechanism that is convened by village committees, deliberated by decision-making committees, and extensively participated by all stakeholders.

Third, the higher the participation ability of residents, the greater the influence of tourism economic empowerment and tourism psychological empowerment on participation willingness. It refers that ethnic villages can enhance residents’ participation ability through providing them skills training to participate in tourism services. Many residents engaged in the tourism industry have begun to awaken their commodity awareness, but sometimes they will inevitably deviate from the direction in understanding the problems. For example, in the construction of family hotels, residents are told that they should try their best to keep the characteristics of traditional dwellings, which can be transformed from old houses. The maintenance of such traditional features often wins the favor of tourists. It is also necessary to train the reception staff and village guides. By broadening the financing channels for poverty alleviation and development, improving the tourism infrastructure and social security system, the residents’ participation ability can be enhanced and the obstacles for the poor with low participation ability can be reduced. With the improvement of participation ability, the space of community participation in tourism will be expanded.

Under the guidance and help of the government, consulting institutions, enterprises, the community plays multiple roles such as adviser, planner, executor, manager, supervisor, and beneficiary. They will be fully aware of the value of their own national culture, have an understanding of the development mechanism of tourism, stimulate their own participation potential, and realize the diversified economic, social and cultural development of the community. In addition, according to the specific situation of the residents, we should also set up a card file, treat residents with different participation ability differently, and implement targeted support and guidance measures to ensure household policies. For example, for residents who are far away from the scenic spot, factories for processing tourist commodities such as ethnic costumes and accessories may be built. For young people who can sing and dance, national cultural song and dance teams can be formed. Through effective anti-poverty assistance mechanism and green participation channel, the disadvantaged groups are ensured to have more opportunities to participate and share the tourism revenue.

The participation ability of village residents in tourism poverty alleviation does not play a moderating role between tourism social empowerment and the willingness to participate, indicating that traditional community organizations such as the old village organizations still play an important role in the process of poverty alleviation through tourism in ethnic villages. Although traditional community organizations are independent of administrative power, to some extent, they can play an irreplaceable role in solving the governance crisis of ethnic villages. They have a strong binding force on villagers, and they are indispensable to mediate when villagers encounter problems or disputes in production and life. In the process of tourism poverty alleviation, traditional community organizations should be endowed with some new functions and play their organizing and coordinating role in the process of tourism poverty alleviation. Traditional community organizations can coordinate to establish ethnic village tourism.

The participation ability of villagers in tourism poverty alleviation does not play a regulating role between tourism political empowerment and the willingness to participate, which indicates that under the background of targeted poverty alleviation, the administrative execution ability of grassroots organizations such as ethnic villages’ village committees has been enhanced. Village committees are an extension of grassroots government organizations. They should play a greater role in tourism poverty alleviation. They should also take the guidance of tourism poverty alleviation as an opportunity to conduct extensive operation and management mobilization, which will not only promote the effect of tourism poverty alleviation, but also enhance the cohesion of grassroots organizations and improve the credibility of the government.

### Limitations and Future Research

There are some limitations to this study. To begin with, although this study follows strict requirements, whether the conclusions drawn from this study that only takes Zenlei Village as a case are applicable to other ethnic villages remains to be further studied. The following scholars can select other ethnic villages to test the external validity of the findings. Moreover, this study proves that ethnic village residents’ participation willingness plays a partial mediating role between tourism empowerment and their participation behavior, there might be other mediating mechanisms between them, subsequent research can adopt other theories to further explore the transmission mechanism. Finally, this study confirms that tourism empowerment can promote ethnic village residents’ participation behavior, yet whether the negative effects of tourism poverty alleviation (e.g., the peaceful lifestyle is disturbed, local traditional notions are changed or even distorted, and so forth) might hinder their participation behavior. If it can, the mediating mechanism and boundary conditions are still unclear, need to be further verified.

## Conclusion

Drawing on planned behavior theory and capacity approach theory, we took Zenlei Village as an example and investigated whether and how tourism empowerment affects ethnic village residents’ behavior to participate in tourism poverty alleviation. The following conclusions are drawn. First, tourism empowerment has a remarkable positive influence on ethnic village residents’ behavior to participate in tourism poverty alleviation. Second, the influence of tourism empowerment on ethnic village residents’ behavior to participate in tourism poverty alleviation could be achieved through the partial mediation of participation willingness. Last, participation ability positively moderate the relationships between tourism economic empowerment, tourism psychological empowerment, and participation willingness. That is, the positive relationships between tourism economic empowerment, tourism psychological empowerment, and participation willingness would be stronger when village residents’ participation ability is higher, yet the relationships between tourism social empowerment, tourism political empowerment, and participation willingness are not affected by participation ability.

## Data Availability Statement

The raw data supporting the conclusions of this article will be made available by the authors, without undue reservation.

## Ethics Statement

The studies involving human participants were reviewed and approved by Academic Committee of Guizhou University of Finance and Economics. Written informed consent to participate in this study was provided by the participants’ legal guardian/next of kin.

## Author Contributions

JY conceived the study and wrote the manuscript. JW wrote and revised the manuscript. LZ analyzed the data. XX conceived the study and revised the manuscript. All authors contributed to the article and approved the submitted version.

## Conflict of Interest

The authors declare that the research was conducted in the absence of any commercial or financial relationships that could be construed as a potential conflict of interest.
